# Anti–Receptor Activator of Nuclear Factor-κB Ligand Improves Muscle Dysfunction and Strengthens Bone in Laminin-α2–Deficient *dy*^*2J*^/*dy*^*2J*^ Dystrophic Mice

**DOI:** 10.1016/j.ajpath.2026.04.011

**Published:** 2026-05-11

**Authors:** Zineb Bouredji, Anthony Facchin, Dounia Hamoudi, Hideo Yagita, Norbert Laroche, Marthe Rousseau, Anteneh Argaw, Jérôme Frenette

**Affiliations:** ∗ Centre Hospitalier Universitaire de Québec–Université Laval Research Center, Axe Neurosciences, Université Laval, Quebec City, Quebec, Canada; † Department of Immunology, School of Medicine, Juntendo University, Tokyo, Japan; ‡ Jean Monnet Université Saint-Étienne, INSERM, Mines Saint Étienne, Santé Ingénierie Biologie St-Étienne U1059, Saint-Étienne, France; § Unité Mixte de Recherche 5510 Matériaux Ingienrie et Science, Centre National de Recherche Scientifique/Lyon University/Institut National de Science Appliquée–Lyon, Lyon, France; ¶ School of Rehabilitation Sciences, Faculty of Medicine, Université Laval, Quebec City, Quebec, Canada

## Abstract

Congenital muscular dystrophy type 1A (CMD1A) is a severe genetic neuromuscular disorder caused by mutations in the *LAMA2* gene, which encodes the laminin-α2 subunit, an essential structural protein for maintaining muscle integrity during contraction. Affected children experience progressive muscle damage, hypotonia, delayed motor development, and respiratory tract insufficiency. CMD1A muscles undergo repeated cycles of degeneration and regeneration accompanied by chronic inflammation. Beyond its established role in bone remodeling, the receptor activator of nuclear factor-κB/receptor activator of nuclear factor-κB ligand (RANKL)/osteoprotegerin signaling axis has also been implicated in muscle pathophysiology. A 10-day treatment with full-length osteoprotegerin fused to an Fc fragment was previously shown to restore muscle function in a Duchenne muscular dystrophy mouse model. In this study, anti-RANKL therapy was evaluated in a more severe mouse model, specifically 4-week–old male *dy*^*2J*^/*dy*^*2J*^ mice, which recapitulate key CMD1A features. A 1-month anti-RANKL treatment significantly improved specific muscle strength, increased myofiber cross-sectional area, and reduced edema and immune cell infiltration. It also enhanced bone strength and microarchitecture. However, absolute muscle force and global motor performance remained unchanged. Overall, anti-RANKL treatment improved muscle intrinsic properties and bone parameters in *dy*^*2J*^/*dy*^*2J*^ dystrophic mice, but these improvements were insufficient to enhance absolute muscle force or global motor performance.

## Graphical abstract

**Figure:**
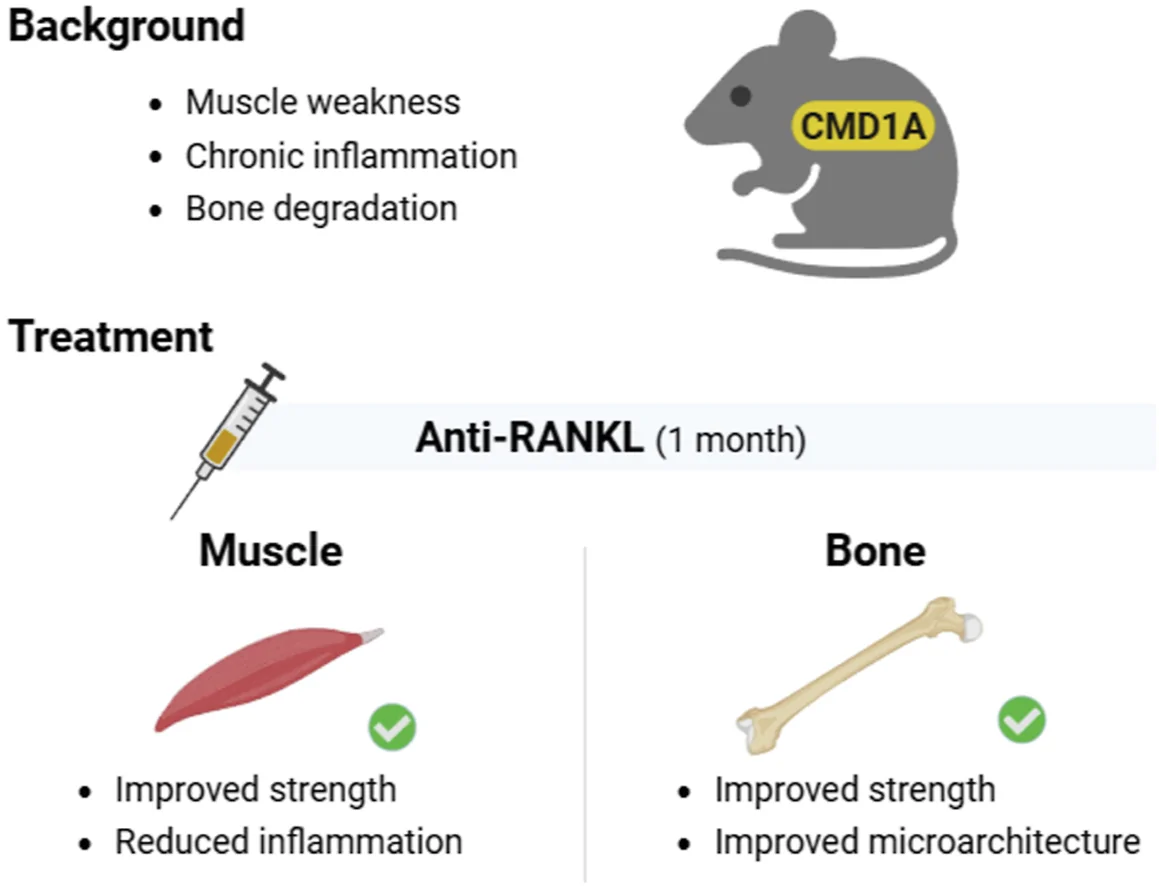

Congenital muscular dystrophy type 1A (CMD1A) is a severe genetic neuromuscular disease caused by mutations in the *LAMA2* gene, which encodes the laminin-α2 subunit. With an estimated prevalence ranging from 1 in 30,000 to 100,000 and accounting for 24% to 37% of the cases, CMD1A is the most common form of human congenital muscular dystrophy in humans.^1^ Laminins are heterotrimeric structural proteins crucial for cell assembly, anchoring the sarcolemma of muscle fibers to the extracellular matrix, thereby ensuring muscle integrity during contractions.^2^ Laminin-211, composed of the *α*2, *β*1, and *γ*1 isoforms, is a major component of the basement membrane of skeletal muscles^3^ and is also expressed in other tissues, such as peripheral nerves (Schwann cells), heart, skin, and brain.^4^ The severity of the disease largely depends on whether the expression of laminin chain is reduced or totally absent.^3^^,^^5^^,^^6^ Patients with CMD1A experience hypotonia, contractures, muscle atrophy, and weakness, accompanied by a marked proliferation of connective tissue.^3^ Their muscles undergo successive cycles of degeneration and regeneration characterized by fiber size heterogeneity, chronic inflammation, and muscle damage, with elevated levels of serum creatine kinase, particularly in the first months of life.^3^ These patients also experience significant motor skill delays and are often wheelchair bound early in life.^3^ The *dy*^*2J*^/*dy*^*2J*^ mouse model is widely used for studying CMD1A, as it expresses a truncated laminin-α2 subunit chain and exhibits a milder muscle phenotype compared with other models.^7^^,^^8^ These mice have a lifespan exceeding 6 months and display key pathologic features observed in patients with CMD1A, including muscle fiber size variability, necrosis, apoptosis, and inflammatory cell infiltration.^8^ RNA sequencing of 8-week–old *dy*^*2J*^/*dy*^*2J*^ skeletal muscle revealed activation of signaling pathways involved in inflammation, apoptosis, and necrosis. Notably, NF-κB, renin-angiotensin, epithelial-mesenchymal transition, and calcium signaling pathways were up-regulated, whereas paired box 7 (Pax7) expression was significantly down-regulated.^9^ Additionally, pathways involved in muscle degradation and autophagy lysosome function^10^ were overactivated, contributing to muscle dysfunction in *dy*^*2J*^/*dy*^*2J*^ mice.^11^

Over the past decade, growing evidence has shown that muscle and bone dysfunctions often occur simultaneously in many pathologic conditions, driven by mutual crosstalk involving cytokines and signaling pathways. Previously, the role of the receptor activator of nuclear factor-κB ligand (RANKL) and receptor activator of nuclear factor-κB (RANK) in Duchenne muscular dystrophy was investigated.^12^, ^13^, ^14^ A sixfold increase in RANK mRNA expression was reported in *mdx* mice (dystrophin-deficient mouse model of Duchenne muscular dystrophy), and muscle-specific deletion of *RANK* was shown to significantly improve skeletal muscle specific force.^14^ In addition, RANK and RANKL protein levels were found to be elevated in the myofiber microenvironment of dystrophin-deficient *mdx* mice. Consistently, 1-month anti-RANKL treatment improved skeletal muscle function by reducing edema, fibrosis, and serum creatine kinase levels, a marker of muscle damage.^12^ In bone, the interaction between RANKL and RANK activates NF-κB, leading to the formation of mature multinucleated osteoclasts and bone resorption.^15^ More recent evidence demonstrates that RANKL neutralization significantly mitigates left ventricular hypertrophy, reduces heart mass, and preserves cardiac function in dystrophic *mdx* mice by inhibiting NF-κB and improving Ca^2+^ homeostasis.^13^ Given the proven benefits of anti-RANKL treatment in improving muscle function, the present study investigated its effects in a more severe and genetically distinct dystrophic mouse model. The results confirm that *dy*^*2J*^/*dy*^*2J*^ mice experience severe muscle dysfunction,^11^ which is independent of sex. Additionally, bone abnormalities and increased RANK expression were observed in *dy*^*2J*^/*dy*^*2J*^ dystrophic muscles. Notably, RANKL inhibition significantly improves the force of dystrophic fast-twitch extensor digitorum longus (EDL) muscle and enhances bone mechanical properties compared with IgG-treated dystrophic mice. Anti-RANKL treatments also reduce excessive leukocyte infiltration in dystrophic muscles. However, these treatments did not translate into significant improvements in locomotor activity, which primarily relies on slow-twitch muscle fibers.

## Materials and Methods

### Animals

All animal experiments were approved by the Université Laval Research Center Animal Care and Use Committee based on the Canadian Council on Animal Care guidelines. Male and female *B6.WK-Lama2dy-*^*2J/J*^ (*dy*^*2J*^) heterozygous couples were initially obtained from Dr. Ronald Cohn's laboratory (University of Toronto, Toronto, ON, Canada) and were back-crossed and bred in a specific pathogen-free animal facility. The mice were housed under a 12:12-hour light/dark cycle with food and water *ad libitum*. The genotypes were determined by PCR amplification and sequencing using the following primers: forward: 5′-GGAAAAAGGACCCGAGATGT-3′; and reverse: 5′-GGATTCATAAAACGCTTACAGG-3′. The 1- and 2-month–old homozygous male and female *dy*^*2J*^/*dy*^*2J*^ mice and their littermate wild-type (WT) mice were used for skeletal muscle and bone analyses. One-month–old male homozygous *dy*^*2J*^/*dy*^*2J*^ mice received nine i.p. injections of 4 mg/kg of mouse monoclonal antibodies targeting RANKL (anti-RANKL; IK22–5) or isotype-matched rat IgG control (Sigma-Aldrich, Oakville, ON, Canada) every 3 days for 4 weeks. All experiments and data acquisition and analyses were performed by blinded experimenters (Z.B., A.F., D.H., and N.L.).

### Open Field Behavioral Activity Measurements

Voluntary locomotor activities of WT and anti-RANKL or IgG-treated *dy*^*2J*^/*dy*^*2J*^ mice were measured using the ANY-maze video tracking system (Stoelting Co, Wood Dale, IL) in a 50 × 50-cm open field for 12 hours starting at 6 pm. The cumulative distance traveled (in meters) was recorded.

### Skeletal Muscle Contractile Properties

The mice were injected subcutaneously with buprenorphine (0.1 mg/kg) and were anesthetized by an i.p. injection of pentobarbital sodium (50 mg/kg). The EDL and soleus (Sol) muscles were gently removed, incubated at 25°C in Krebs-Ringer physiological solution (pH 7.4), supplemented with 95% O_2_ and 5% CO_2_, and attached to an electrode and a force sensor (305B-LR; Aurora Scientific, Aurora, ON, Canada). Contractile properties were measured using a 305B-LR dual-mode muscle arm system monitored by a dynamic muscle data acquisition and analysis system (Aurora Scientific). Maximum specific tetanic tension sP_0_ (N/cm^2^) values were obtained by normalizing the absolute force P_0_ to the fiber cross-sectional area (CSA) using the following equation: sP_0_ = P_0_/CSA. CSA was determined by dividing the muscle mass by the product of the optimum fiber length corresponding to the result of multiplying optimal length with the fiber length ratio (0.44 and 0.71 for EDL and Sol muscles, respectively) and the muscle density (1.06 mg/mm^3^). Muscle contractility results were analyzed using Dynamic Muscle Data Analysis software V5.300 (Aurora Scientific).

### Bone Mechanical Properties

Bone functional properties were evaluated by a three-point binding test at middiaphysis using an MTS Bionix servohydraulic test system (MTS Systems Corp., Eden Prairie, MN). The tibiae from 1- and 2-month–old male homozygous *dy*^*2J*^/*dy*^*2J*^ mice and their WT controls were isolated and placed on a set of supports 1 cm apart. The rate of displacement was 2 mm/minute until failure. The load displacement curve for each bone was analyzed, and the mechanical properties, such as ultimate load and stiffness, were recorded as described previously.^16^

### Bone Microarchitecture Analysis

Femur bones were scanned *ex vivo* using micro–computed tomography (VivaCT40; Scanco, Brüttisellen, Switzerland) with 70 peak kilovoltage under a resolution of 10.5 μm. Segmentation parameters for reconstruction and trabecular analyses were 0.8/1/260 for cortical bone. Only cortical bone marrow area (mm^2^), cortical porosity (%), bone area (mm^2^), and cortical thickness (mm) were evaluated.

### Dry and Wet Muscle Mass Measurements

The EDL muscles from IgG- or anti–RANKL-treated male *dy*^*2J*^/*dy*^*2J*^ mice were weighed to determine their wet mass, and they were dried overnight at 65°C, and weighed again to determine the dry mass. The percentage of water content was calculated using the following equation: 100 × (wet mass – dry mass)/wet mass.

### Immunohistochemistry

Cross-sections of EDL muscles from WT, IgG-treated, or anti–RANKL-treated *dy*^*2J*^/*dy*^*2J*^ mice were washed with phosphate-buffered saline, fixed for 10 minutes with 4% paraformaldehyde, blocked with 5% bovine serum albumin and 1% horse serum solution, and then incubated overnight at 4°C with the following primary antibodies: anti–Ly6-G/C (BD Bioscience, Mississauga, ON, Canada; 1:300), anti-laminin (Sigma-Aldrich; 1:250), and anti-RANK (Abcam, Cambridge, UK; 1:200) diluted in blocking solution. The sections were washed three times with phosphate-buffered saline and were incubated with Alexa Fluor 488– or 594–conjugated secondary antibodies (Invitrogen, Burlington, ON, Canada; 1:500) for 2 hours at room temperature. The sections were washed three times and mounted with DAPI Fluoromount-G (SouthernBiotech, Birmingham, AL). Immunofluorescence labeling was analyzed using an Axio Imager M2 microscope (Zeiss, Oberkochen, Germany) connected to an AxioCam camera using ZEN2 software blue edition (Zeiss). CSA of the fibers was measured from laminin-DAPI immunofluorescence labeling using ImageJ software version 1.53t (NIH, Bethesda, MD; *https://imagej.nih.gov/ij*), and the variance coefficient of CSA was calculated using the Feret diameter method, as follows: variance coefficient = (SD of the muscle fiber size/mean muscle fiber size) × 1000.^17^ Neutrophil infiltration was quantified from at least four fields acquired using a 20× objective, and data are presented as cell density (number of cells/mm^3^).

### Western Blot Analysis

Frozen muscles were homogenized for 45 seconds on ice in radioimmunoprecipitation assay lysis buffer supplemented with a cocktail of protease inhibitors (P8340; Sigma-Aldrich) and phosphatase inhibitors (2 mmol/L Na_3_VO_4_, 50 mmol/L NaF, and 0.2 mmol/L phenylmethylsulfonyl fluoride; Sigma-Aldrich) using a tissue homogenizer (PowerGen 125; ThermoFisher Scientific, Waltham, MA). The protein content was determined using a bicinchoninic acid protein assay kit (EMD Chemical, Ottawa, ON, Canada). The homogenates were heated to 95°C in Laemmli buffer for 2 minutes, separated by SDS-PAGE, and transferred to a polyvinylidene difluoride membrane (Bio-Rad, Hercules, CA). The membranes were blocked in 5% skim milk and were incubated overnight at 4°C with the following primary antibodies: anti-RANK (BAF692; R&D System, Toronto, ON, Canada; 1:500), anti–phosphorylated NF-κB (3033; Cell Signaling, Whitby, ON, Canada; 1:500), anti–NF-κB (4764S; Cell Signaling; 1:1000), and anti–muscle RING-finger 1 (MuRF1) (ab183094; Abcam; 1:1000). The membranes were washed, incubated with the appropriate horseradish peroxidase–conjugated secondary antibodies (1:10,000), and visualized by chemiluminescence using the ECL-Plus imaging system (Perkin-Elmer, Woodbridge, ON, Canada). Band densities were analyzed using Quantity One software version 4.6.6 (Bio-Rad, Mississauga, ON, Canada) and were normalized to the total protein content.

### Statistical Analysis

All values are expressed as means ± SEM. The data were analyzed using either *t*-test or Dunnett *post hoc* test (one- or two-way analysis of variance) using Prism version 3.1 (GraphPad Software, San Diego, CA). The levels of significance were set at ∗*P* < 0.05, ∗∗*P* < 0.01, ∗∗∗*P* < 0.001, and ∗∗∗∗*P* < 0.0001.

## Results

### Laminin-α2 Chain Deficiency Impairs Muscle and Bone Function in Both 1- and 2-Month–Old Male and Female *dy*^*2J*^/*dy*^*2J*^ Mice

**Figure 1:**
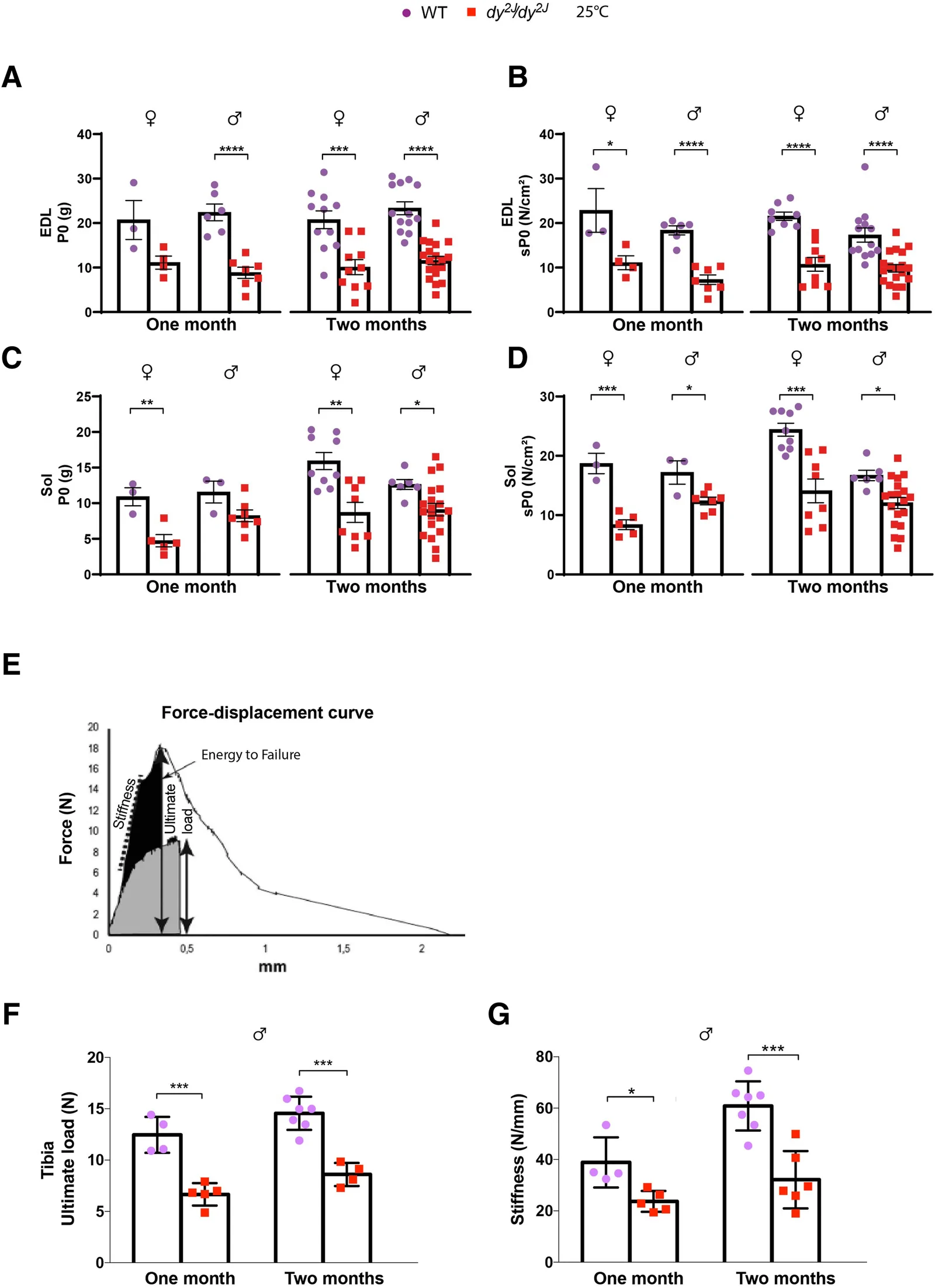
Laminin-α2 chain deficiency impairs muscle function and bone mechanical properties in *dy*^*2J*^/*dy*^*2J*^ mice. *Ex vivo* analysis of contractile properties was performed on fast-twitch extensor digitorum longus (EDL) and slow-twitch soleus (Sol) muscles from 1- and 2-month–old male and female wild-type (WT) and *dy*^*2J*^/*dy*^*2J*^ mice. Both maximum isometric (P0) and specific forces (sP0) were significantly reduced in the EDL (**A** and **B**) and Sol (**C** and **D**) muscles of 2-month–old *dy*^*2J*^/*dy*^*2J*^ mice compared with age- and sex-matched WT controls. To assess bone mechanical integrity, a three-point bending test was conducted *ex vivo* at middiaphysis on tibiae isolated from male WT and *dy*^*2J*^/*dy*^*2J*^ mice. The resulting force-displacement curve (**E**) revealed significantly lower ultimate load (**F**) and stiffness (**G**) in *dy*^*2J*^/*dy*^*2J*^ mice at both 1 and 2 months of age, indicating compromised bone strength relative to WT counterparts. Data are expressed as means ± SEM (**A**–**D**, **F**, and **G**). *n* = 3 to 19 (**A**–**D**, **F**, and **G**). ∗*P* < 0.05, ∗∗*P* < 0.01, ∗∗∗*P* < 0.001, and ∗∗∗∗*P* < 0.0001 (significantly different from the age-matched WT using *t*-test).

Although several studies have reported muscle dysfunction in *dy*^*2J*^/*dy*^*2J*^ dystrophic mice,^11^^,^^18^, ^19^, ^20^ data on sex-based differences remain limited. In this study, the *ex vivo* contractile properties of fast-twitch EDL and slow-twitch Sol muscles were evaluated in 1- and 2-month–old female and male *dy*^*2J*^/*dy*^*2J*^ mice and their age-matched WT littermates. Both female and male *dy*^*2J*^/*dy*^*2J*^ mice exhibited significant reductions (approximately 50%) in absolute and specific forces of EDL muscles at both ages (Figure 1, A and B). In Sol muscles, absolute force was significantly reduced in both sexes at 1 month of age compared with WT controls (Figure 1C). At 2 months, male *dy*^*2J*^/*dy*^*2J*^ mice showed a modest but significant reduction in Sol muscle force, whereas female mice displayed a more pronounced decline (Figure 1C). Specific force of Sol muscles was significantly lower in both sexes at both time points (Figure 1D). Body mass was significantly lower in *dy*^*2J*^/*dy*^*2J*^ mice compared with WT controls at both ages and sexes (Table 1). At 2 months, EDL muscle mass was significantly reduced in both sexes, whereas Sol muscle mass remained unchanged. Optimal fiber length of EDL muscles was significantly shorter in *dy*^*2J*^/*dy*^*2J*^ males at both ages and 2-month–old *dy*^*2J*^/*dy*^*2J*^ females (Table 1). Optimal length of Sol muscles remain unchanged (Table 1). Given the comparable muscle impairments observed in both sexes, subsequent bone function analyses focused on male mice. A three-point bending test was performed *ex vivo* on tibiae from WT and *dy*^*2J*^/*dy*^*2J*^ males generating force-displacement curves (Figure 1E). Both ultimate load (Figure 1F) and stiffness (Figure 1G) were significantly reduced in *dy*^*2J*^/*dy*^*2J*^ males at 1 and 2 months of age compared with WT controls.

**Table 1:** Body Mass and Morphologic Characteristics of EDL and Sol Muscles in 1- and 2-Month–Old Male and Female *dy*^*2J*^/*dy*^*2J*^ Mice and Age-Matched WT Controls

Group	Mouse mass, means ± SEM, g	EDL, means ± SEM		Sol, means ± SEM	
		Muscle mass, mg	L_0_, mm	Muscle mass, mg	L_0_, mm
WT 1-month–old female	20.67 ± 2.60	5.2 ± 0.12	12.33 ± 0.61	5.03 ± 0.69	11.37 ± 0.82
*dy*^*2J*^*/dy*^*2J*^ 1-Month–old female	15 ± 0.32∗	5.6 ± 0.23	11.91 ± 0.45	4.06 ± 0.26	10.57 ± 0.30
WT 1-month–old male	22.67 ± 0.92	7.2 ± 0.54	12.6 ± 0.35	5.6 ± 0.25	11.10 ± 0.35
*dy*^*2J*^*/dy*^*2J*^ 1-Month–old male	19.14 ± 0.55∗∗	6.3 ± 0.21	11.12 ± 0.29∗∗	5.24 ± 0.41	10.84 ± 0.18
WT 2-month–old female	19.7 ± 0.60	6.9 ± 0.41	13.3 ± 0.29	5.8 ± 0.25	11.99 ± 0.18
*dy*^*2J*^*/dy*^*2J*^ 2-Month–old female	17.79 ± 0.52∗	5.97 ± 0.18∗	12.41 ± 0.17∗	5.53 ± 0.33	12.02 ± 0.18
WT 2-month–old male	24.08 ± 0.65	8.68 ± 0.35	13.36 ± 0.16	7.3 ± 0.21	12.83 ± 0.48
*dy*^*2J*^*/dy*^*2J*^ 2-Month–old male	20.35 ± 0.35∗∗∗∗	7.03 ± 0.20∗∗∗	12.66 ± 0.22∗	6.83 ± 0.32	12.23 ± 0.16

EDL and Sol muscles were incubated *ex vivo* in a controlled physiological environment (Krebs-Ringer supplemented with 95% O_2_ and 5% CO_2_, pH 7.4) at 25°C. Muscle L_0_ and mass were determined. Body mass was significantly lower in both 1- and 2-month–old *dy*^*2J*^/*dy*^*2J*^ mice compared with age- and sex-matched WT counterparts. EDL muscle mass was significantly reduced in 2-month–old male and female *dy*^*2J*^/*dy*^*2J*^ mice relative to WT. L_0_ values of EDL muscles were significantly lower in both 1-and 2-month–old *dy*^*2J*^/*dy*^*2J*^ males compared with WT. This reduction is also observed in 2-month–old *dy*^*2J*^/*dy*^*2J*^ females compared with age-matched WT controls. For Sol muscles, there were no statistical differences in muscle mass or L_0_ in *dy*^*2J*^/*dy*^*2J*^ mice compared with age- or sex-paired WT controls. Data are expressed as means ± SEM. *n* = 3 to 20.

∗*P* < 0.05, ∗∗*P* < 0.01, ∗∗∗*P* < 0.001, and ∗∗∗∗*P* < 0.0001 (significantly different from WT mice using the *t*-test).

EDL, extensor digitorum longus; L_0_, optimal length; Sol, soleus; WT, wild type.

**Figure 2:**
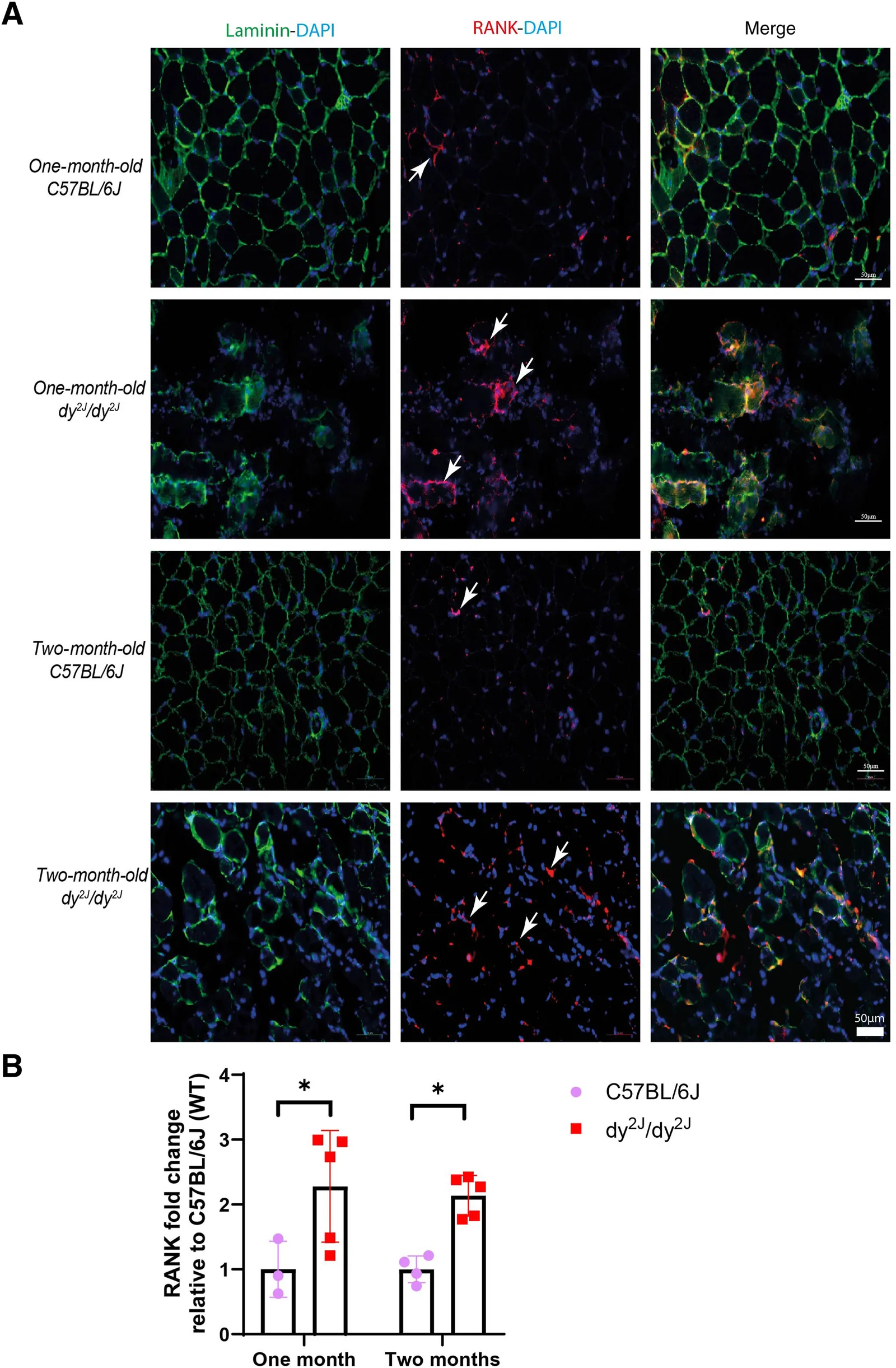
Elevated RANK expression in skeletal muscle of 1- and 2-month–old male *dy*^*2J*^/*dy*^*2J*^ mice. **A:** Immunohistochemical analysis of extensor digitorum longus muscles from 1- and 2-month–old male *dy*^*2J*^/*dy*^*2J*^ mice and their age-matched wild-type (WT) mice revealed increased expression of RANK (red; indicated by **white arrows**), with costaining for laminin (green) and DAPI (blue). **A** and **B:** RANK expression was markedly elevated in *dy*^*2J*^/*dy*^*2J*^ muscle tissue compared with WT controls at both time points. *n* = 3 to 5 (**B**). ∗*P* < 0.05 (significantly different from the age-matched WT using Kruskal-Wallis test, followed by Dunn *post hoc* test). Scale bar = 50 μm (**A**).

Elevated RANK expression was previously reported in the dystrophic skeletal muscle microenvironment of *mdx* mice.^14^ In the present study, RANK protein levels were assessed in EDL muscles from male *dy*^*2J*^/*dy*^*2J*^ and WT male mice at 1 and 2 months of age by immunofluorescence staining. Colabeling for RANK, laminin, and nuclei revealed markedly increased RANK expression in both 1- and 2-month–old *dy*^*2J*^/*dy*^*2J*^ males compared with WT controls (Figure 2).

### Anti-RANKL Treatment Enhances EDL Muscle-Specific Force and Tibial Bone Properties in Dystrophic *dy*^*2J*^/*dy*^*2J*^ Mice

**Figure 3:**
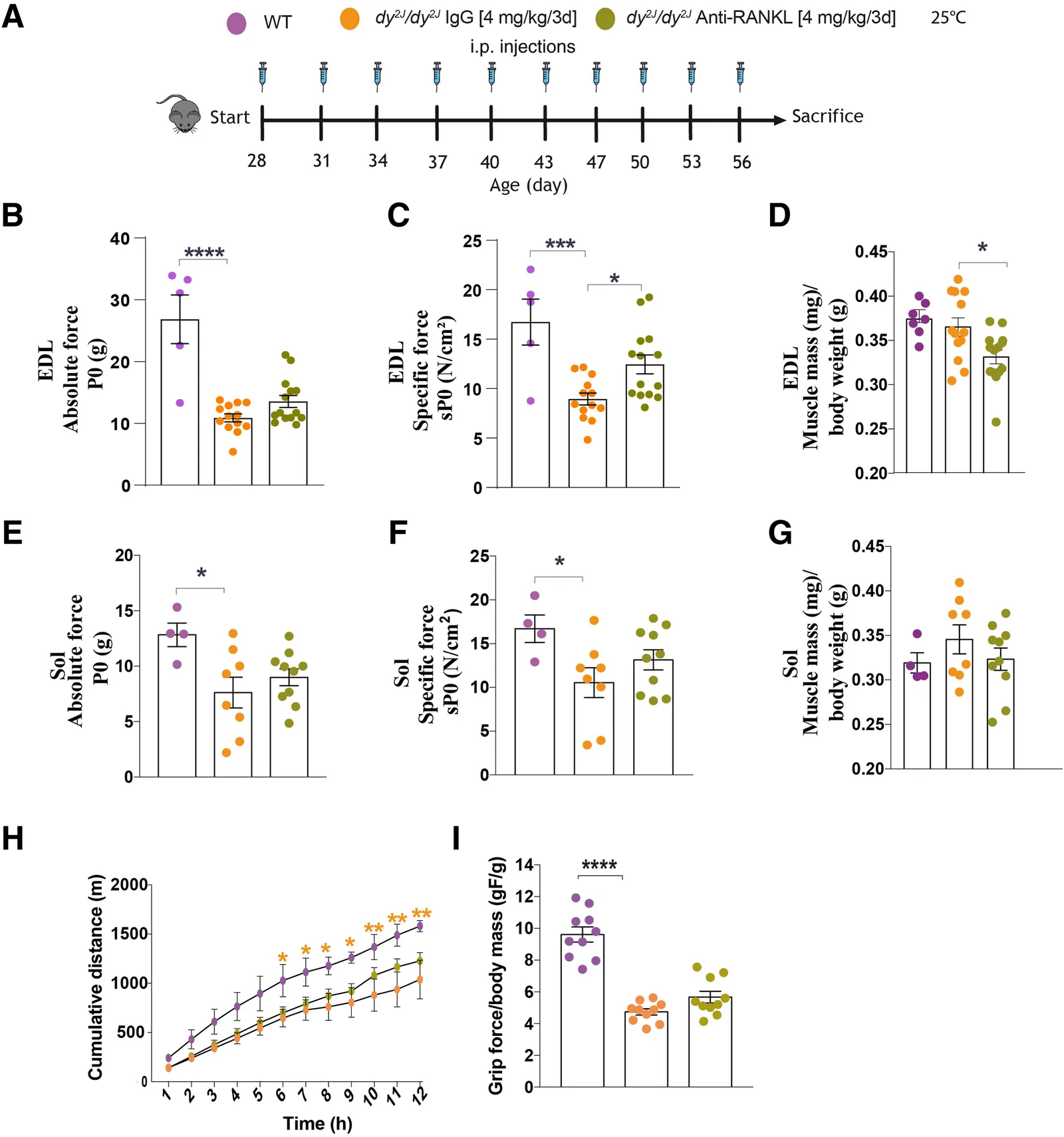
Anti-RANKL treatment enhances specific force in fast-twitch extensor digitorum longus (EDL) muscles but has limited functional impact in *dy*^*2J*^/*dy*^*2J*^ mice. **A:***Ex vivo* assessment of contractile properties was performed on the fast-twitch (EDL) and slow-twitch soleus (Sol) muscles from 1-month–old male *dy*^*2J*^/*dy*^*2J*^ mice treated for 28 days with either control IgG or anti-RANKL (4 mg/kg per 3 days). **B**, **C**, and **E:** Anti-RANKL treatment did not significantly improve the absolute isometric force (P0) in either EDL or Sol muscles (**B** and **E**, respectively); however, it significantly increased the specific force (sP0) of EDL muscle (**C**). **D:** Anti-RANKL reduced EDL muscle mass/body weight ratio. **F** and **G:** No significant changes were observed in Sol muscle contractile properties between treatment groups. **H:** Voluntary locomotor activity, measured using the ANY-maze video tracking system, did not show significant differences in cumulative distance traveled between the anti–RANKL- and IgG-treated mice. **I:** Similarly, no difference was observed in grip strength normalized to body weight between the anti-RANKL and the IgG groups. Data are expressed as means ± SEM (**B**–**I**). *n* = 4 to 13 (**B**–**I**). ∗*P* < 0.05, ∗∗*P* < 0.01, ∗∗∗*P* < 0.001, and ∗∗∗∗*P* < 0.0001 (significantly different from IgG-treated *dy*^*2J*^/*dy*^*2J*^ mice using one- or two-way analysis of variance with Bonferroni multiple-comparison test). WT, wild type.

To investigate the effects of inhibiting the RANKL/RANK interaction, 1-month–old male *dy*^*2J*^/*dy*^*2J*^ mice received i.p. injections of anti-mouse RANKL (4 mg/kg per 3 days for 28 days (Figure 3A). Following treatment, the contractile properties of the EDL and Sol muscles were assessed *ex vivo*. Anti-RANKL treatment did not increase absolute tetanic force (Figure 3B) but significantly improved the maximum specific force of dystrophic EDL muscles (8.9 ± 0.6 versus 12.5 ± 1.0 N/cm^2^) (Figure 3C). Additionally, the EDL muscle mass/body weight ratio was reduced in anti–RANKL-treated mice compared with IgG control *dy*^*2J*^/*dy*^*2J*^ mice (0.37 ± 0.01 versus 0.33 ± 0.01) (Figure 3D), suggesting a potential reduction in edema or muscle hypertrophy. In contrast, anti-RANKL treatment had no significant effect on the absolute or specific force, nor on the muscle mass/body weight ratio, of Sol muscles compared with IgG-treated *dy*^*2J*^/*dy*^*2J*^ mice (Figure 3, E–G). Although voluntary cage activity over 12 hours (Figure 3H) or grip strength normalized to body weight (Figure 3I) showed slight improvements in the anti-RANKL group, these changes were not statistically significant (*P* = 0.15).

**Figure 4:**
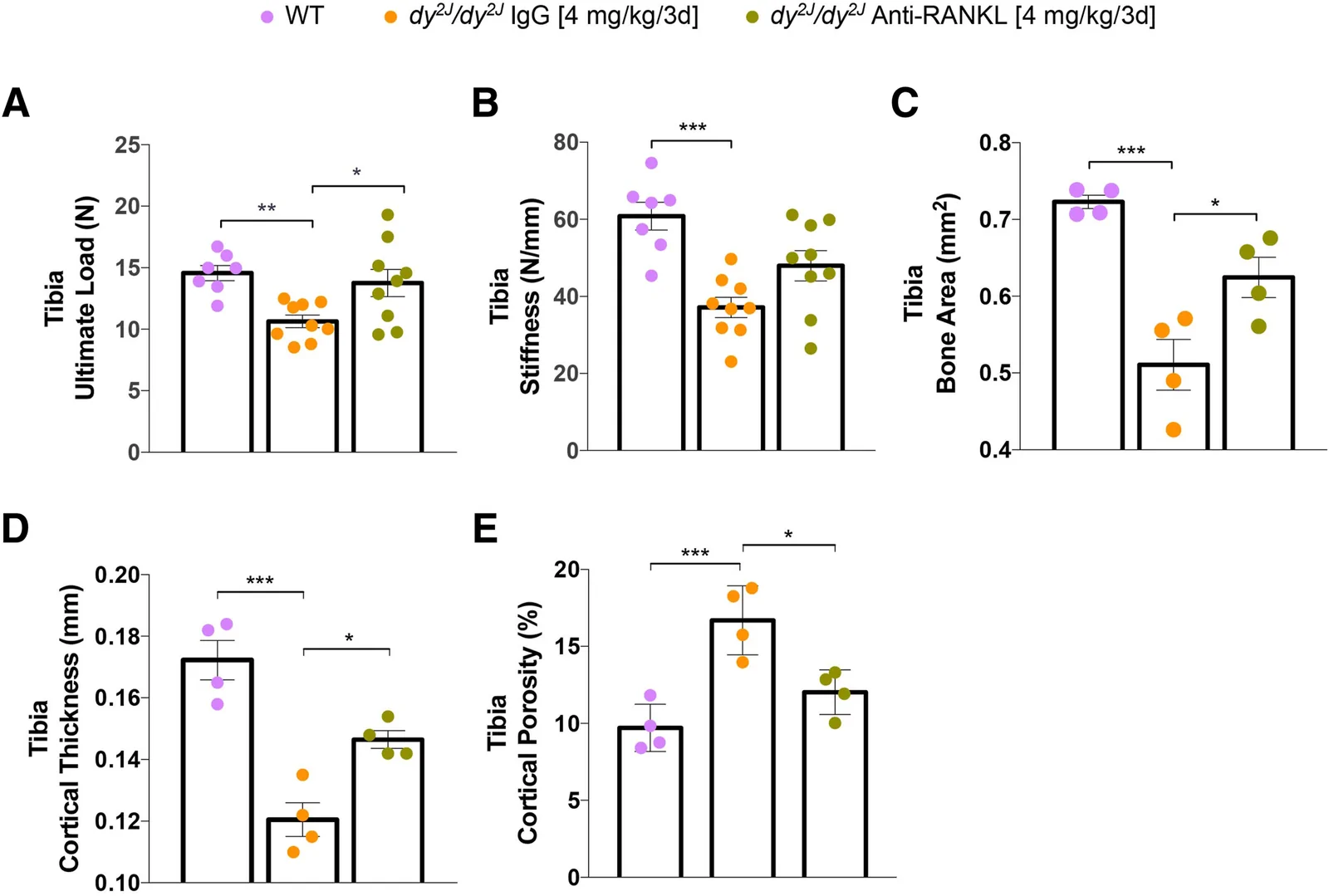
Anti-RANKL treatment improves bone mechanical properties and cortical microarchitecture in *dy*^*2J*^/*dy*^*2J*^ mice. *Ex vivo* three-point bending tests were performed at middiaphysis of tibiae from male *dy*^*2J*^/*dy*^*2J*^ mice treated with either control IgG or anti-RANKL (4 mg/kg per 3 days). **A:** Anti-RANKL treatment significantly increased ultimate load, indicating improved bone mechanical strength. **B:** However, tibia stiffness showed a trend toward significance following anti-RANKL treatment but did not reach statistical significance. **C**–**E:** Micro–computed tomography analysis of tibia cortical bone revealed that anti-RANKL treatment significantly enhanced cortical bone area (**C**) and cortical thickness (**D**) while reducing cortical porosity (**E**), compared with IgG-treated controls. These findings suggest that anti-RANKL therapy improves both the structural and mechanical integrity of bone dystrophic mice. Data are expressed as means ± SEM (**A**–**E**). *n* = 4 to 9 (**A**–**E**). ∗*P* < 0.05, ∗∗*P* < 0.01, and ∗∗∗*P* < 0.001 (significantly different from IgG-treated *dy*^*2J*^/*dy*^*2J*^ mice using an analysis of variance with Bonferroni multiple-comparison test). WT, wild type.

To assess bone integrity, a three-point bending test at the middiaphysis was performed *ex vivo* on tibiae isolated from treated mice. Anti-RANKL treatment significantly increased the bone's ultimate load (Figure 4A) and showed a trend toward increased stiffness (*P* = 0.06) (Figure 4B) in anti–RANKL-treated *dy*^*2J*^/*dy*^*2J*^ mice compared with the IgG-treated controls. Microarchitectural analysis further revealed significant improvements in bone area (0.58 ± 0.04 versus 0.69 ± 0.07 mm^2^) (Figure 4C) and the cortical thickness (0.14 ± 0.0 versus 0.16 ± 0.0 mm) (Figure 4D), along with a significant reduction in cortical porosity (11.9% ± 0.8% versus 9.3% ± 0.9%) (Figure 4E).

### Anti-RANKL Treatment Enhances Muscle Integrity in *dy*^*2J*^/*dy*^*2J*^ Mice

**Figure 5:**
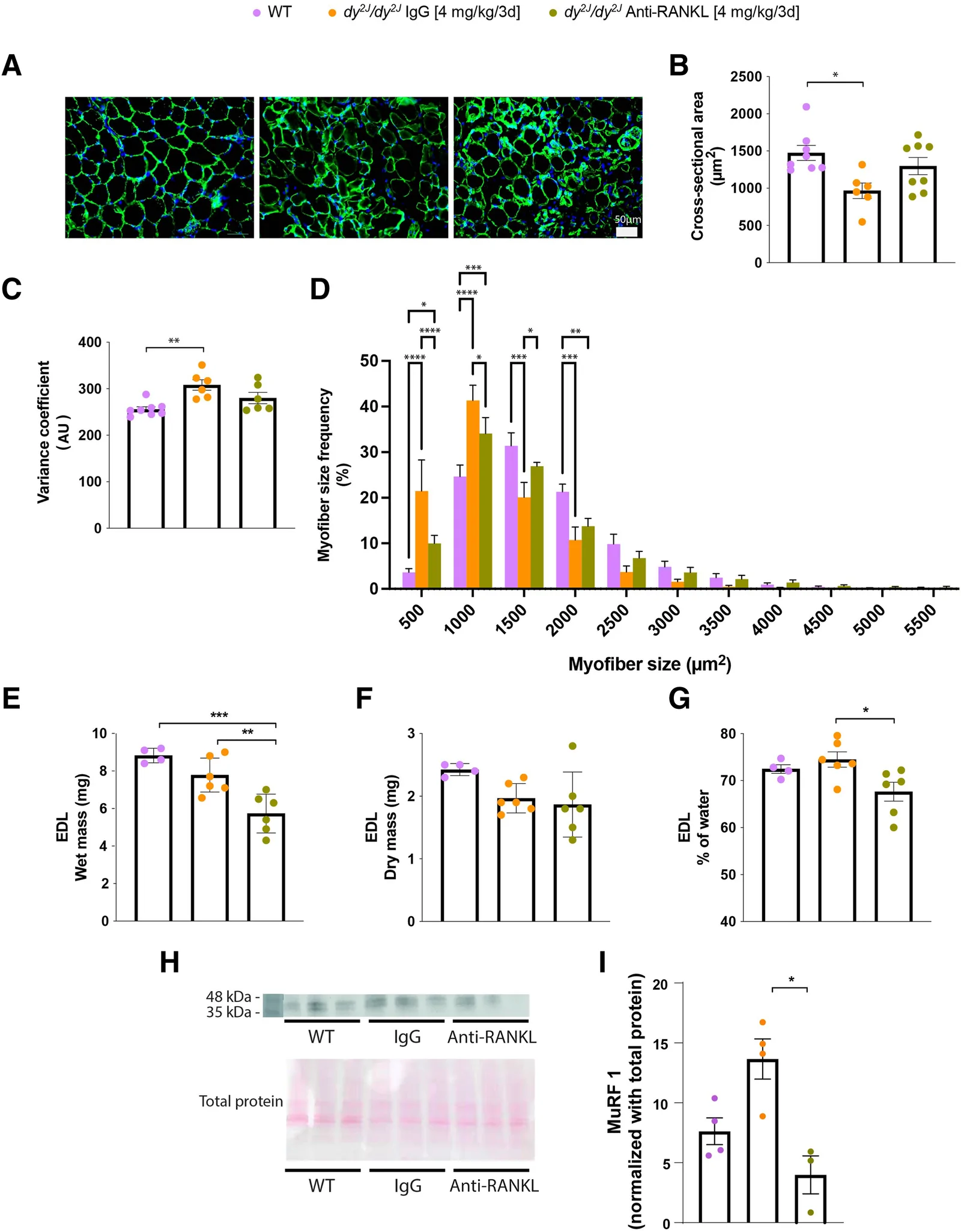
Anti-RANKL treatment modulates myofiber size and reduces wet mass and water content in extensor digitorum longus (EDL) muscles of *dy*^*2J*^/*dy*^*2J*^ mice. **A:** Cross-sections of EDL muscles from *dy*^*2J*^/*dy*^*2J*^ mice treated for 28 days with either anti-RANKL (4 mg/kg per 3 days) or control IgG for 28 days were immunolabeled with anti-laminin (green) and DAPI (blue). **B:** Myofibers from IgG-treated *dy*^*2J*^/*dy*^*2J*^ mice exhibited significantly reduced cross-sectional area (CSA) compared with wild-type (WT) controls. **B:** Anti-RANKL treatment modestly increased CSA in dystrophic EDL muscles relative to IgG-treated counterparts. **C:** Quantification of the myofiber size heterogeneity using the CV of CSA, based on minimal Feret diameter, revealed significantly greater variability in IgG-treated *dy*^*2J*^/*dy*^*2J*^ mice compared with WT. **D:** Frequency distribution analysis of myofiber CSA showed a shift toward medium-sized myofibers and a significant reduction in small-sized myofibers following anti-RANKL treatment. To assess muscle hydration, EDL muscles were weighed before and after overnight drying to determine wet mass, dry mass, and percentage water content. **E**–**G:** Anti-RANKL treatment significantly reduced both wet mass (**E**) and water content (**G**), whereas dry mass remained unchanged (**F**), suggesting a reduction in muscle edema without loss of muscle tissue. **H** and **I:** Western blot analysis of muscle RING-finger 1 (MuRF1) protein levels in EDL muscles from *dy*^*2J*^/*dy*^*2J*^ mice treated with anti-RANKL or IgG (**H**) revealed a significant reduction in MuRF1 expression following anti-RANKL treatment (**I**). Data are expressed as means ± SEM (**B**–**G** and **I**). *n* = 3 to 8 (**B–G**). ∗*P* < 0.05, ∗∗*P* < 0.01, ∗∗∗*P* < 0.001, and ∗∗∗∗*P* < 0.0001 (significantly different from IgG-treated mice using analysis of variance with Bonferroni multiple-comparison test). Scale bar = 50 μm (**A**). AU, arbitrary unit.

Anti-RANKL treatment was previously shown to promote a shift in fiber size distribution and reduce edema in dystrophic *mdx* mice.^12^ In the present study, the effects of a 28-day anti-RANKL regimen on muscle integrity were examined in *dy*^*2J*^/*dy*^*2J*^ mice. Analysis of the CSA of EDL muscles revealed that IgG-treated mice exhibited significantly smaller myofibers compared with WT controls (Figure 5, A and B). The CV, calculated via the minimal Feret diameter to assess fiber size heterogeneity, was markedly elevated in IgG-treated *dy*^*2J*^/*dy*^*2J*^ EDL muscles relative to WT (308.0 ± 11.4 versus 255.9 ± 5.2 arbitrary units) (Figure 5C). Although anti-RANKL treatment modestly reduced this variability Figure 5C), the change was not statistically significant. CSA frequency distribution analysis further revealed a significant reduction in the proportion of small myofibers (≤1000 μm^2^) and a corresponding increase in medium-sized fibers (approximately1500 μm^2^) following anti-RANKL treatment (Figure 5D). Notably, anti-RANKL administration resulted in a >25% reduction in EDL muscle wet mass (Figure 5E), without affecting dry muscle mass (Figure 5F), suggesting a decrease in muscle edema. This interpretation is supported by an approximately 10% reduction in the wet mass/body weight ratio in treated mice compared with IgG controls (Figure 5G). Western blot analysis revealed a significant reduction in MuRF1 levels in anti–RANKL-treated muscles compared with IgG-treated controls (4.0 ± 1.6 versus 13.6 ± 1.7 arbitrary units) (Figure 5, H and I), consistent with reduced muscle atrophy and improved muscle integrity.

### An Anti-RANKL Treatment Significantly Reduces Neutrophil and Macrophage Infiltration in *dy*^*2J*^/*dy*^*2J*^ Dystrophic EDL Muscles

**Figure 6:**
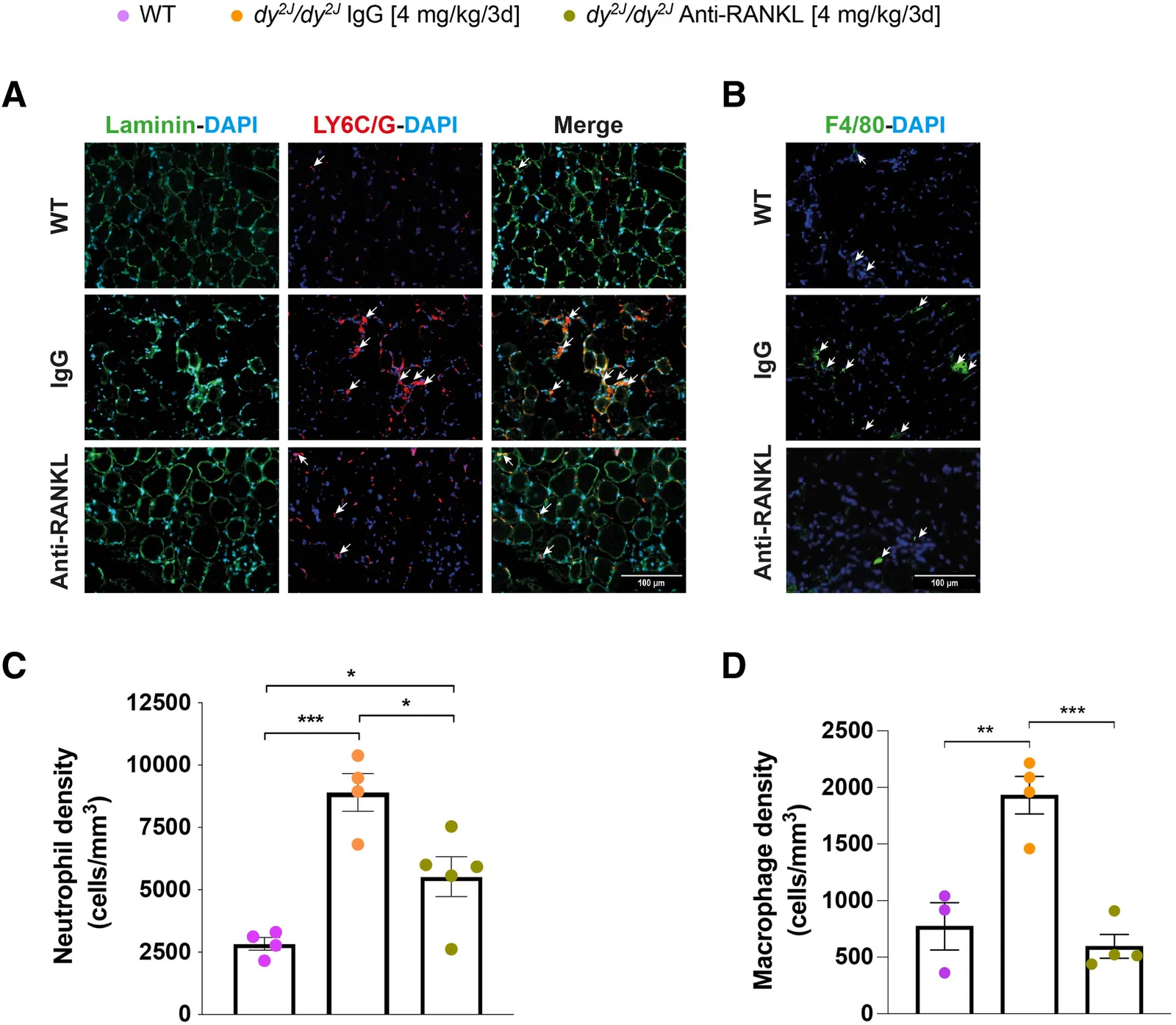
Anti-RANKL treatment reduces neutrophil and macrophage infiltration in extensor digitorum longus (EDL) muscles of *dy*^*2J*^/*dy*^*2J*^ mice. **A** and **B:** Cross-sections of EDL muscles from *dy*^*2J*^/*dy*^*2J*^ mice treated with either anti-RANKL or control IgG were immunolabeled with anti–Ly6-G/C (red; neutrophil marker identified by **white arrows**), anti-laminin (green), and DAPI (blue; **A**) and anti-F4/80 (green; macrophage marker identified by **white arrows**) and DAPI (blue; **B**). **C:** Neutrophil density was significantly elevated approximately threefold in IgG-treated dystrophic muscles compared with wild-type (WT) controls, whereas anti-RANKL treatment reduced neutrophil infiltration by 40%. **D:** IgG-treated *dy*^*2J*^/*dy*^*2J*^ muscles exhibited a significantly increased presence of macrophages, which was markedly reduced following anti-RANKL treatment. These findings indicate that anti-RANKL therapy attenuates inflammatory cell infiltration in dystrophic muscle tissue. Data are expressed as means ± SEM (**C** and **D**). *n* = 3 to 5 (**C** and **D**). ∗*P* < 0.05, ∗∗*P* < 0.01, and ∗∗∗*P* < 0.001 (significantly different from IgG-treated mice using analysis of variance with Bonferroni multiple-comparison test). Scale bar = 100 μm (**A** and **B**).

Given the significant reduction in wet muscle mass following anti-RANKL treatment (Figure 5E), together with the shift in fiber size distribution toward a more WT-like phenotype (Figure 5D), it was hypothesized that this treatment regimen mitigates excessive edema and inflammatory cell infiltration. To test this hypothesis, neutrophil and macrophage infiltration in EDL muscle was assessed by immunofluorescence. Quantification of Ly6G/C, a marker of neutrophils, revealed significantly elevated neutrophil presence in IgG-treated *dy*^*2J*^/*dy*^*2J*^ muscles compared with WT controls (Figure 6, A and C). Anti-RANKL treatment significantly reduced neutrophil infiltration (Figure 6, A and C). Similarly, macrophage density was markedly higher in IgG-treated muscles relative to WT controls. Anti-RANKL treatment significantly decreased macrophage infiltration as well (Figure 6, B and D), further supporting its anti-inflammatory effect in dystrophic muscle tissue.

## Discussion

Children affected by CMD1A exhibit profound hypotonia, muscle weakness, delayed motor development, feeding difficulties, joint contractures, progressive respiratory tract insufficiency, and cardiomyopathy.^5^ Currently, there is no cure for CMD1A, and most affected individuals do not survive beyond adolescence. The laminin-α2–deficient *dy*^*2J*^/*dy*^*2J*^ mouse is the most widely used rodent model for merosin-deficient congenital muscular dystrophy type 1A. These mice exhibit hallmark features of the disease, including skeletal muscle fiber atrophy, inflammation, and increased fibrosis.^11^ RANKL neutralization was previously shown to improve both bone and muscle function in *mdx* mice, a murine model of Duchenne muscular dystrophy.^12^^,^^14^ Given that laminin-α2 deficiency, like Duchenne muscular dystrophy, compromises sarcolemmal integrity, the current study examined whether anti-RANKL treatment could mitigate skeletal muscle dysfunction in *dy*^*2J*^/*dy*^*2J*^ mice, a more severe and genetically different dystrophic mouse model. These findings show that *dy*^*2J*^/*dy*^*2J*^ mice exhibit severe muscle and bone abnormalities independent of sex. Notably, RANK is overexpressed in skeletal muscles of these dystrophic mice. Inhibiting RANKL significantly improved dystrophic EDL muscle force, enhanced muscle integrity, and strengthened bone mechanical properties. Additionally, anti-RANKL treatment reduced edema and attenuated excessive neutrophil and macrophage infiltration, highlighting its potential as a therapeutic strategy for merosin-deficient congenital muscular dystrophy type 1A.

Although limb muscle functions are well characterized in both male and female *dy*^*2J*^/*dy*^*2J*^ mice across different ages and diseases,^11^ data comparing sex-related differences in the contractile properties of fast- and slow-twitch muscle phenotypes remain lacking. In this study, isolated muscle function was first assessed in 1- and 2-month–old male and female *dy*^*2J*^/*dy*^*2J*^ mice. Consistent with findings by Bressler et al,^18^ both fast-twitch EDL and slow-twitch Sol muscles from *dy*^*2J*^/*dy*^*2J*^ mice exhibited significantly reduced isometric and specific forces compared with WT mice. These results further confirm that there are no sex-related difference in the contractile properties of these muscle types, aligning with previous observations on limb muscle function.

Emerging evidence suggests that the co-occurrence of osteoporosis and muscle atrophy arises from shared molecular mechanisms affecting both tissues in various pathologic conditions.^21^, ^22^, ^23^ In line with this, the study demonstrates, for the first time, that *dy*^*2J*^/*dy*^*2J*^ mice exhibited severe impairments in bone mechanical properties, as evidenced by significant reductions in bone ultimate load and stiffness. Ontogenetic allometry analyses further support a link between bone size and disease severity: *dy*/*dy* mice, which present a more severe dystrophic phenotype than *dy*^*2J*^/*dy*^*2J*^ mice, also display pronounced bone atrophy.^24^ Similarly significant bone loss has also been reported in both male and female *mdx* mice,^25^ reinforcing the concept of musculoskeletal deterioration across different forms of muscular dystrophy. Although reduced skeletal muscle force generation likely contributes to bone remodeling and resorption, local and systemic cytokines secreted by dystrophic muscle may also drive bone loss in *dy*^*2J*^/*dy*^*2J*^ mice.^25^ The RANK/RANKL/osteoprotegerin signaling pathway emerges as a potential mechanistic link between muscle pathology and bone fragility in this model. Previous studies have shown that RANKL and its receptor RANK are up-regulated in the dystrophic muscle microenvironement, promoting bone resorption and influencing both bone and muscle function.^12^ Although the role of growth factors or cytokines in regulating muscle and bone mass in patients with CMD1A and animal models remains to be fully elucidated, current evidence strongly suggests that the RANK/RANKL/osteoprotegerin triad is involved in the regulation of musculoskeletal atrophy in muscular dystrophy.^12^

Because RANK signaling contributes to dystrophic muscle dysfunction, the present study examined whether neutralizing RANKL could improve skeletal muscle contractility in laminin-*α*2–deficient (*dy*^*2J*^/*dy*^*2J*^) mice. These findings demonstrate that anti-RANKL treatment significantly enhances both muscle and bone strength in this model. These results build on previous work in *mdx* mice, where a 1-month anti-RANKL regimen improved skeletal muscle function and bone mechanical properties.^12^ More recently, anti-RANKL treatment was also shown to prevent glucocorticoid-induced bone loss by preserving bone structure and strength, while simultaneously improving muscle integrity through reduced neutrophil infiltration, decreased fibrosis, and enhanced muscle fiber organization.^26^ In the current study, anti-RANKL treatment led to a reduction in the number of small, atrophic fibers (<500 μm^2^ CSA) and an increase in the density of larger myofibers (>1500 μm^2^), suggesting a shift toward a more regenerative or anabolic muscle phenotype. These findings imply that RANKL inhibition may suppress catabolic signaling and/or promote muscle regeneration. Supporting this, prior studies demonstrated that RANKL stimulation of C2C12 myotubes up-regulates the expression of atrogin-1 and MuRF1, key mediators of muscle protein degradation and atrophy.^16^ The results of the present study further support that inhibition of RANKL/RANK interaction is associated with reduced MuRF1 expression, consistent with preservation of muscle mass and structural integrity. Similarly, Bonnet et al^27^ reported that RANKL overexpression in muscle induces inflammation, necrosis, and atrophy, whereas its inhibition ameliorates muscle pathology in a transgenic mouse model. Notably, the same study showed that a 3-year treatment with denosumab, a monoclonal antibody targeting RANKL, improved appendicular lean mass and handgrip strength in postmenopausal women.^27^ Despite these positive effects, anti-RANKL did not improve voluntary locomotor activity in *dy*^*2J*^/*dy*^*2J*^ mice. By 4 weeks of age, these mice already exhibit extensive muscle damage, degeneration, and fibrosis,^11^ suggesting that intervention at this stage may be insufficient to reverse the established pathology. Moreover, laminin-α2 deficiency also affects peripheral nerves, leading to Schwann cell demyelination and early-onset neuropathy, which contributes to hind limb paralysis of the inferior members at the early stage of the disease.^28^^,^^29^ It is important to underline that *ex vivo* muscle stimulation bypasses dysfunctional motoneurons, allowing for direct and complete assessment of muscle contractility. Last, previous studies with full-length osteoprotegerin fused to an Fc fragment and anti-RANKL consistently show greater protective effects in fast-twitch compared with slow-twitch dystrophic myofibers.^14^ Because slow-twitch myofibers are predominantly recruited during voluntary locomotion, this fiber-type specificity may limit the observable impact of RANKL inhibition on whole-body motor performance. Together, these factors may help explain the discrepancy between improved isolated muscle function and the lack of improvement in global locomotion activity in *dy*^*2J*^/*dy*^*2J*^ mice.

In the present study, a marked increase in inflammatory cell infiltration was observed in the EDL muscle of *dy*^*2J*^/*dy*^*2J*^ mice compared with WT controls. Treatment with anti-RANKL significantly reduced the presence of both neutrophils and macrophages in dystrophic muscles at 2 months of age. These findings are consistent with previous studies demonstrating that anti-RANKL treatment reduces inflammation in the EDL muscles of *mdx* mice.^12^^,^^26^ RANKL, a member of the tumor necrosis factor superfamily, modulates immune response through a TNF receptor-associated factor (TRAF) and NF-κB signaling pathway.^30^ In earlier work, the ratio of phosphorylated NF-κB/total NF-κB was shown to be elevated in dystrophic muscles and normalized following anti-RANKL treatment in *mdx* mice.^12^ This suggests that RANKL contributes to NF-κB activation and the associated proinflammatory environment in dystrophic muscle, a mechanism likely conserved in *dy*^*2J*^/*dy*^*2J*^ mice. Furthermore, Carbone et al^31^ demonstrated that RANKL promotes neutrophil migration and degranulation in the infracted heart, effects that were reversed by anti-RANKL treatment, further highlighting its role in modulating inflammation and tissue damage repair. A recent publication also confirmed that anti-RANKL reduces neutrophil infiltration in dystrophic muscle of mdx mice.^26^ Although the data clearly show a reduced immune cell infiltration following anti-RANKL treatment, it remains to be determined whether this effect is attributable to a direct inhibition of neutrophil and macrophage recruitment or an indirect consequence of reduced muscle degeneration and regeneration cycles. Nonetheless, the involvement of RANKL in NF-κB pathway activation has been well established *in vitro* and *in vivo* in skeletal and cardiac muscle contexts.^12^^,^^13^ Furthermore, transcriptomic profiling and pathway enrichment analyses of *dy*^*2J*^/*dy*^*2J*^ skeletal muscle revealed up-regulation of pathways associated with inflammation, fibrosis, cell migration, apoptosis, necrosis, NF-κB activation, and calcium signaling, alongside down-regulation of key myogenic markers Pax7, Pax3, and MEF2-activating motif and SAP domain-containing transcriptional regulator (Mamstr).^9^ Moreover, calcium uptake into the sarcoplasmic reticulum is impaired in both fast- and slow-twitch muscles of *dy*^*2J*^/*dy*^*2J*^ mice because of reduced sarcoplasmic/endoplasmic reticulum Ca^2+^ ATPase levels.^32^ Previous work demonstrated that anti-RANKL restores sarcoplasmic/endoplasmic reticulum Ca^2+^ ATPase activity and sarcoplasmic/endoplasmic reticulum Ca^2+^ ATPase-2a expression in fast-twitch dystrophic skeletal and cardiac muscles.^13^^,^^14^ These findings suggest that the muscle weakness and muscle wasting observed in *dy*^*2J*^/*dy*^*2J*^ mice may result from a combination of inflammatory signaling, which potentially disrupts degeneration/regeneration dynamics and impairs calcium handling, all of which may be ameliorated by anti-RANKL treatment through improved cytosolic calcium homeostasis.

In summary, *dy*^*2J*^/*dy*^*2J*^ mice exhibit profound musculoskeletal impairments, characterized by significant reductions in skeletal muscle force production and compromised bone stiffness and stress resistance. This study is the first to show that RANK protein is highly expressed in the skeletal muscles of *dy*^*2J*^*/dy*^*2J*^ mice. Importantly, pharmacologic inhibition of the RANKL-RANK interaction, achieved through anti-RANKL treatment, leads to notable improvements in muscle contractile function, reductions in muscle edema and immune cell infiltration, and enhancements in bone function and microarchitecture. However, given that *dy*^*2J*^/*dy*^*2J*^ mice already showed substantial muscle weakness by 4 weeks of age, the therapeutic window for effective intervention may be narrow. These findings suggest that the benefits of anti-RANKL treatment could be limited when initiated after the onset of significant pathology. Therefore, future studies may explore the efficacy of earlier intervention ideally at or before weaning, to determine whether initiating treatment during the critical development window can yield more robust and sustained functional improvements.

## Declaration of Generative AI and AI-Assisted Technologies in the Writing Process

At the end of the preparation of this work, the authors used Copilot AI (Microsoft, Redmond, WA) to assist with syntax verification and orthographic revision. After using this tool/service, the authors reviewed and edited the content as needed and take full responsibility for the content of the publication.

## Disclosure Statement

None declared.
